# Raman scattering of InAs/AlAs quantum dot superlattices grown on (001) and (311)B GaAs surfaces

**DOI:** 10.1186/1556-276X-7-476

**Published:** 2012-08-23

**Authors:** Alexander Milekhin, Nikolay Yeryukov, Alexander Toropov, Dmitry Dmitriev, Evgeniya Sheremet, Dietrich RT Zahn

**Affiliations:** 1 Institute of Semiconductor Physics, Lavrentjevav 13, Novosibirsk 630090, Russia; 2 Novosibirsk State University, Pirogovstr 2, Novosibirsk 630090, Russia; 3 Semiconductor Physics, Chemnitz University of Technology, Chemnitz, D-09107, Germany

**Keywords:** Raman scattering spectroscopy, Quantum dots, Nanocrystals, Nanoparticles, Phonons

## Abstract

We present a comparative analysis of Raman scattering by acoustic and optical phonons in InAs/AlAs quantum dot superlattices grown on (001) and (311)B GaAs surfaces. Doublets of folded longitudinal acoustic phonons up to the fifth order were observed in the Raman spectra of (001)- and (311)B-oriented quantum dot superlattices measured in polarized scattering geometries. The energy positions of the folded acoustic phonons are well described by the elastic continuum model. Besides the acoustic phonons, the spectra display features related to confined transverse and longitudinal optical as well as interface phonons in quantum dots and spacer layers. Their frequency positions are discussed in terms of phonon confinement, elastic stress, and atomic intermixing.

## Background

Semiconductor nanostructures such as quantum dot superlattices (QD SLs) grown by molecular beam epitaxy (MBE) in the Stranski-Krastanov growth mode offer unique opportunity of engineering their electron and phonon spectra with the most appropriate properties for nanodevices. Among optical techniques, Raman spectroscopy is considered as the most informative method for determining phonon spectra of semiconductor nanostructures including QD SLs consisting of a variety of materials (Ge/Si, (In,Ga,Al)Sb/GaAs, In(As,Sb)/InP, InAs/(Al,Ga)As) [1-8].

The phonon spectrum of QD SLs presents a fundamental interest because of new physical phenomena (such as localization of optical phonons, interference of acoustic phonons, and spectrum renormalization of interface phonons in QD SLs). It also provides valuable information on structural parameters of QDs such as QD size and shape as well as strain and atomic intermixing in QD structures. Most of the published data related to phonons in QD SLs were obtained by Raman scattering experiments and refer mainly to the study of resonance effects on acoustic and optical phonons in Ge/Si QD SLs [1,2], to establishing the phonon spectra in novel QD SLs [3,4], and to investigation of the topology effects as well as strain and intermixing on the optical and interface phonons in InAs/AlGaAs QD SLs [5-7]. InAs/AlAs QDs are of special interest because they have electronic bandgap transitions in the visible spectral range (600 to 700 nm) making resonant Raman scattering experiments possible [8].

The majority of the reported Raman experiments were carried out for QD SLs grown along the principal crystallographic axis [001]. For these structures, according to the Raman selection rules, only longitudinal optical (LO) phonons can be observed in backscattering from the planar surfaces of the SLs while rather sophisticated scattering geometries should be used for probing the transverse optical (TO) phonons [6]. The QD SLs grown on high-index surfaces present significant interest for optoelectronic applications because they reveal intensive narrow linewidth bandgap photoluminescence. These structures are much less investigated by Raman spectroscopy despite Raman selection rules allow simultaneous observation of both LO and TO phonons in backscattering experiments from the planar surface. Here, we report the comparison of Raman spectra by acoustic and optical phonons in InAs/AlAs QD SL fabricated on (001) and (311)B GaAs surfaces in the same growth process.

## Methods

InAs/AlAs QD SLs were grown by molecular beam epitaxy in a Riber 32P system simultaneously on (001)- and (113)B-oriented GaAs substrates utilizing Stranski-Krastanov growth mode. Samples are composed of 20 periods of InAs QD layers with a nominal thickness of 2.4 monolayers separated by AlAs spacer layers with thicknesses of 6, 8, 10, and 13 nm (samples A_*n*_, B_*n*_, C_*n*_, and D_*n*_, respectively). Here, the indexes *n* = 001 and 113 note the sample growth direction. The substrate temperature was 460°C during the growth of InAs QDs at an arsenic pressure of 8 × 10^−6^Torr. After the deposition of the nominal amount of island material, the growth was interrupted for 10 s for InAs QDs. The growth was monitored by reflection high-energy electron diffraction (RHEED). According to RHEED data, the transition from a two-dimensional to a three-dimensional growth mode (beginning of island formation) for all the samples occurs after the deposition of 1.8 monolayers of the QD material. After the dot formation, the first 5 nm of AlAs spacers were grown at the same temperature as the QDs (460°C). Then the temperature was increased to 610°C, and the rest of the AlAs spacer was deposited.

Raman spectra in acoustic and optical phonons were recorded at *T* = 20 and 300 K using a Dilor XY800 triple spectrometer (HORIBA JobinYvon Inc., Edison, NJ, USA). Different lines of Ar^+^ and Kr^+^ lasers (457.9 to 676.4 nm) were used for excitation. The spectra were measured in backscattering geometries parallel to the growth axes in both polarized (z(xx) − z, z′(x′x′) − z′) and depolarized configurations (z(yx) − z, and z′(y′x′) − z′) with x, y, z, x′, y′, z′ parallel to the [100], [010], [001], [−110], [33–2], and [113] directions, respectively. The spectral resolution was 2 cm^−1^ over the entire spectral range.

## Results and discussion

### Acoustic phonons

**Figure 1:**
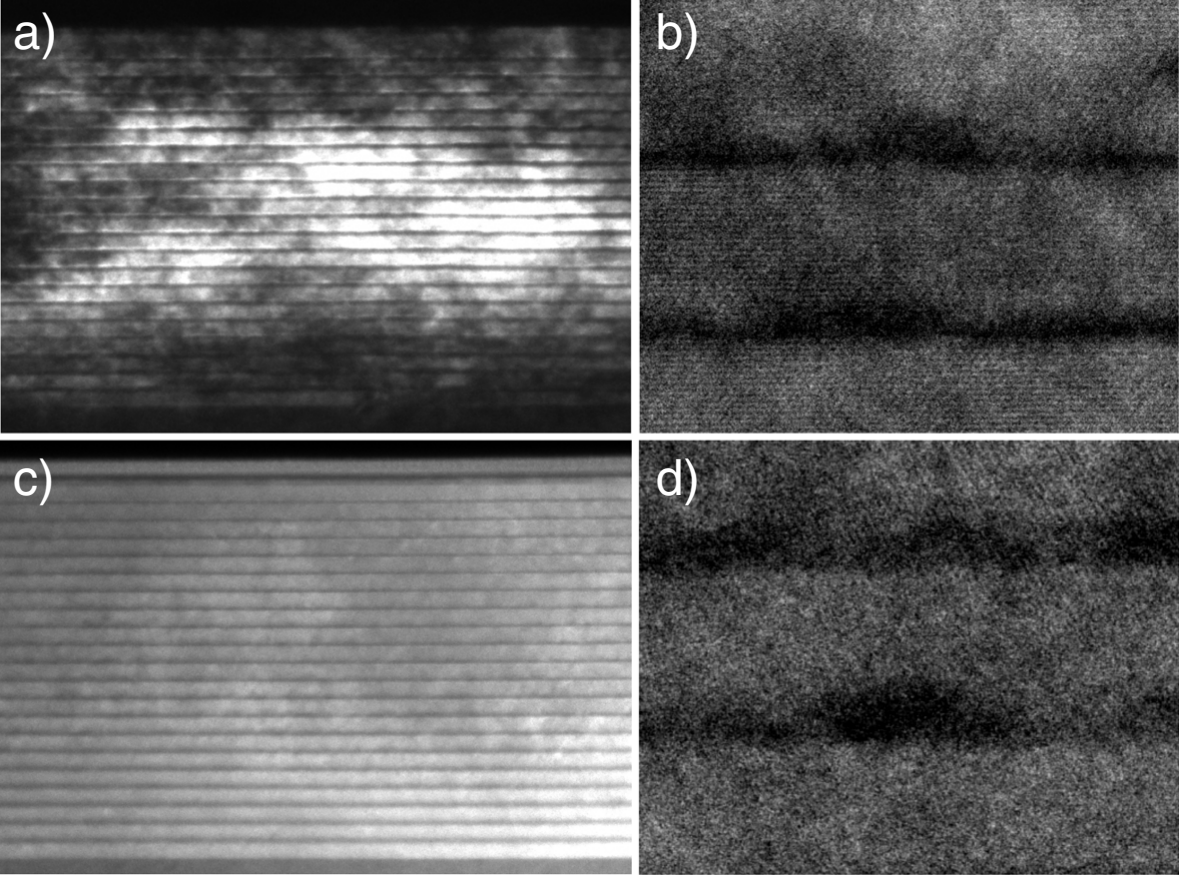
**Cross-sectional TEM and HRTEM images. **Of InAs/AlAs QD SLs grown on (**a**), (**b**) (001)- and (**c**), (**d**) (113)B-oriented GaAs.

The structures under investigation were characterized by high-resolution transmission electron microscopy (HRTEM). Cross-sectional TEM images of samples B_001_ and B_113_ are shown in Figure 1 and reveal highly ordered periodic structures with randomly distributed InAs QDs in the layers. InAs QDs have pyramid-like shape with a base length about 10 to 15 nm and a height of about 1.5 to 2 nm for both substrate orientations. HRTEM images of InAs QD structures demonstrate the presence of an InAs wetting layer. These data are in good agreement with the previously reported ones [6]. 

**Figure 2:**
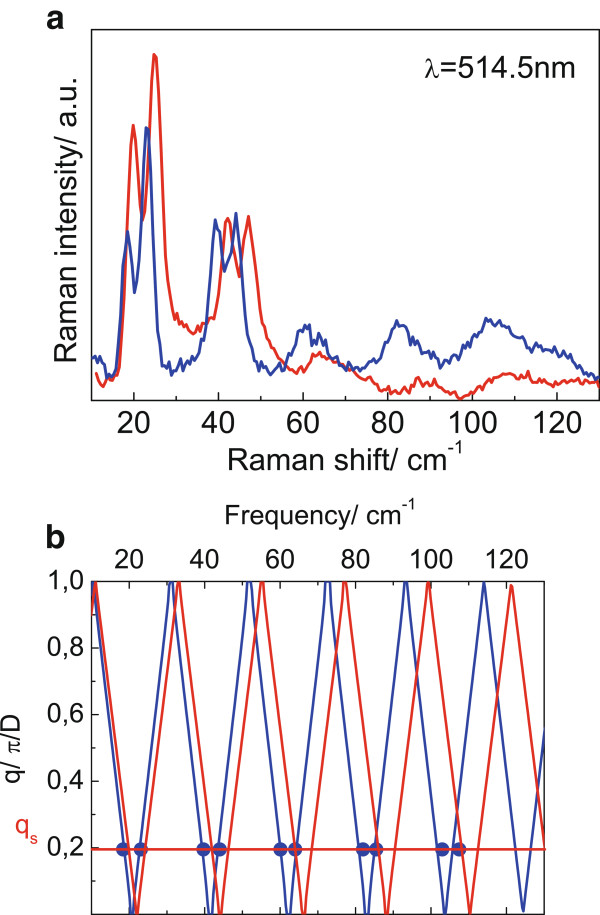
**Raman spectra and dispersion. **(**a**) Raman spectra of samples B_001_ and B_113_ (blue and red curves, respectively) measured in acoustic spectral range with excitation wavelength of 514.5 nm. (**b**) Dispersion of folded LA and QLA phonons calculated for samples B_001_ and B_113_ with nominal thickness (blue and red curves, respectively). A horizontal line and solid blue symbols indicate the value of the scattering wave vector used in the Raman experiment and the frequencies of the folded LA phonons.

**Figure 3:**
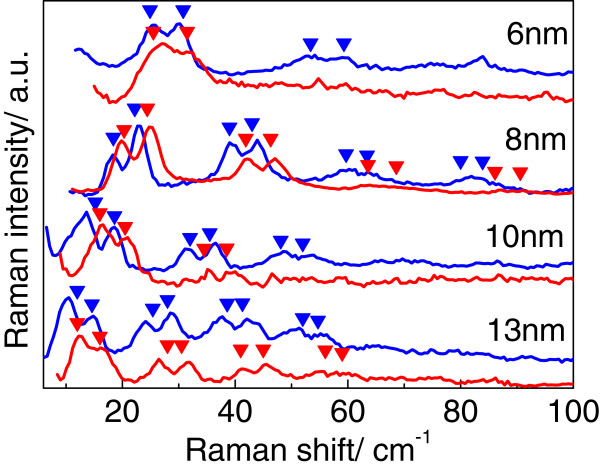
**Raman spectra and the calculated frequency positions. **(**a**) Raman spectra for samples A_001_, B_001_, C_001_, D_001_ and A_113_, C_113_, D_113_ (blue and red curves, respectively) measured in acoustic spectral range with excitation wavelength of 514.5 nm. (**b**) The calculated frequency positions of folded LA and QLA phonons shown by blue and red triangles, respectively.

Figure 2a shows the Raman spectra of samples B_001_ and B_113_ measured in the acoustical spectral range in polarized (z(xx) − z and z′(x′x′) − z′) scattering geometries. In depolarized (z(yx) − z and z′(y′x′) − z′) scattering geometries, Raman spectra are featureless in the acoustic spectral range; therefore, they are not shown in Figure 2. According to the selection rules, Raman spectra of samples B_001_ and B_113_ measured in the polarized geometry should reveal doublets of longitudinal acoustic (LA) and quasi-longitudinal acoustic (QLA) folded phonons. Indeed, doublets of folded acoustic phonons are clearly observed up to the fifth order (Figure 2a). In the case of QD SLs grown in (113)B direction, acoustic phonons propagating along (113) direction have mixed LA-TA character [9]. The sound velocities of QLA and quasi-TA modes were calculated in [9]. Using the elastic continuum model [10], the dispersions of folded LA and QLA phonons were calculated (Figure 2b). The frequency positions of folded LA phonons were determined at the scattering wave vector $q_{s}=\frac{4\pi n}{\lambda_{L}}$, where *λ*_L_ = 514.5 nm is the laser line, and *n* is the refractive index of QD SL at 514.5 nm. The best agreement of calculated and experimental data was obtained for parameters taken from [11]. The period of structure derived from the calculation coincides with the nominal thickness set during the MBE process. An excellent agreement of the experimental and calculated frequency position of the folded LA phonons was also obtained for samples A_001_, C_001_, and D_001_. Dispersion of folded QLA phonons was calculated for samples A_113_, C_113_, and D_113_ taking sound velocities calculated for QLA phonons propagating along the [113] direction [9] into account. The comparison of the frequency positions of the folded acoustic phonons observed in the experiment and calculation using the nominal thickness of QD SLs as a parameter is shown in Figure 3. One can see from Figure 3 that the model of elastic continuum which is usually used for the description of the acoustic spectrum of planar semiconductor SL is well applicable to InAs/AlAs QD SLs grown on (001)- and (113)B-oriented surfaces. 

### Optical phonons

**Figure 4:**
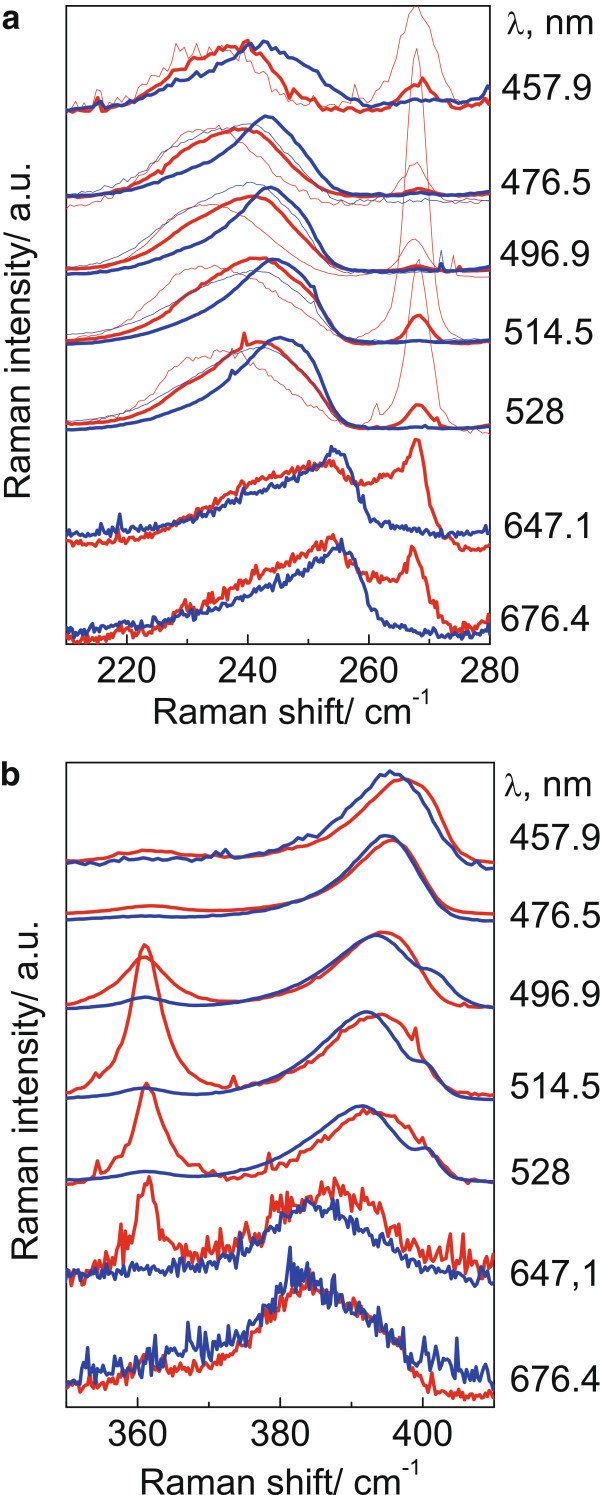
**Raman spectra of samples B **_**001 **_**and B **_**113 **_**(blue and red curves, respectively). **Measured in the spectral ranges of (**a**) InAs measured in both polarized and depolarized (thin lines) geometries and (**b**) AlAs optical phonons measured in the polarized geometry with different excitation wavelengths.

Raman spectra of samples grown on (001)- and (113)B-oriented GaAs were also studied in the spectral range of optical phonons. Figure 4 shows typical Raman spectra (for samples B_001_ and B_113_) measured in the spectral range of InAs and AlAs optical phonons. In the case of sample B_001_, LO phonons in InAs QDs are observed in the polarized geometry. One can see that the feature assigned to LO phonons localized in InAs/AlAs QDs shifts from 255 to 242 cm^−1^ with decrease in the excitation wavelength from 676.4 to 457.9 nm (from 1.83 to 2.71 eV) while the frequency position of TO optical phonons from GaAs (268 cm^−1^) remains unchanged. This behavior of LO phonons in InAs QDs was already explained by QD size distribution and phonon confinement in small-size dots [12,13]. In large QDs, phonon confinement effects are negligible, and the optical phonon energies are affected by strain only. These QDs have lower energies of electronic interband transitions (below 1.8 eV). The contribution of these QDs to the Raman spectra is stronger for excitation lines in the red spectral range that are closer to the resonance with electronic transitions. Therefore, the position of the LO phonon lines of InAs QDs in the Raman spectra excited with 1.83 eV (255 cm^−1^) is determined predominantly by the strain distribution, while the influence of the phonon confinement can be neglected. The smaller QDs have higher energies of interband transitions. Therefore, the relative contribution of small dots to the Raman spectra increases at higher excitation energies. In smaller-size dots, the phonon confinement effect becomes more significant and causes a noticeable decrease in the phonon frequencies (up to 242 cm^−1^). 

According to [13], the In/Al atomic intermixing is relatively small for this growth temperature, and the Al content in InAs QDs is below 15%. As one can see from Figure 3, the behavior of LO phonons as a function of excitation energy in InAs QDs grown on (113)B-oriented GaAs surfaces (sample B_113_) is similar to that observed for InAs QDs on (001) GaAs (sample B_001_). However, the frequency position of the LO phonon in sample B_113_ is regularly lower (on 2 to 5 cm^−1^) than that for sample B_001_. This indicates enhanced In/Al atomic intermixing that leads to increasing Al content in InAs QDs on (113)B GaAs (up to 20%). Raman spectra of InAs QDs grown on (113)B-oriented GaAs measured in depolarized geometry reveal additional features near 230 cm^−1^ attributed TO phonons localized in InAs QDs. These frequency positions of features remain unchanged due to negligible dispersion of TO phonons in InAs [11,13].

Raman spectra of InAs QDs measured in the polarized geometry with different excitation wavelengths show interesting behavior of interface phonons (Figure 4b). In this geometry, AlAs-like interface (IF) phonons localized in the vicinity of InAs QDs are observed. Their frequency positions are located between LO and TO mode frequencies and are shifted from 380 to 395 cm^−1^ with decreasing the excitation wavelength (increasing excitation energy) from 676.4 to 457.9 nm (from 1.83 to 2.71 eV). This behavior can be explained by Raman scattering of InAs QD array having not only different size but also different QD aspect ratio (QD height/base size). It was already shown [5] that the IF phonon frequencies depend on the aspect ratio in InAs/AlAs QDs having oblate shape. In the case of spherical QDs, InAs-like IF phonons have frequencies located in the middle between the frequencies of LO and TO phonons. With decreasing the aspect ratio, the IF phonon frequencies aspire to frequencies of LO phonons in InAs QDs. One can see from Figure 3b that IF phonons in sample B_113_ have higher frequencies at higher excitation energies than that in sample B_001_, thus, indicating lower QD aspect ratio for sample B_001_.

Thus, with increasing excitation energy, InAs QDs having smaller height and larger base size are selectively excited in the Raman process. This is accomplished by the decreasing frequencies of InAs phonons localized in InAs/AlAs QDs and increasing frequencies of IF AlAs-like phonons localized in the vicinity of InAs QDs having lower aspect ratio.

## Conclusions

InAs/AlAs QD SLs were grown on (001)- and (113)B-oriented GaAs with excellent optical and crystalline quality confirmed by high-resolution transmission electron microscopy and Raman scattering by folded acoustical phonons observed up to the fifth order. The Raman spectra were interpreted within the elastic continuum model, and excellent agreement between the measured and calculated data was obtained. The dependences of optical and interface modes in the QD structure on the excitation energies were explained in terms of size and shape selective Raman scattering.

## Competing interests

The authors declare that they have no competing interests.

## Authors’ contributions

All authors read and approved the final manuscript.

## Authors’ information

AM is a senior researcher and Dr. of Science, NY is a senior researcher and Ph.D. student, AT is a doctor and senior researcher, and DD is a researcher at the Institute of Semiconductor Physics, Novosibirsk, Russia. ES is a Ph.D. student and DRTZ is a professor at the Semiconductor Physics, Chemnitz University of Technology, Chemnitz, Germany.
